# Research Progress Review on the Activation of Bioactive Substances by Targeted Fermentation of Rice Bran

**DOI:** 10.3390/foods15111881

**Published:** 2026-05-26

**Authors:** Dong Liang, Chunxu Wei, Dongdong Liu, Xiaofei Liu, Shuangcai Xiao, Yuhao Wang, Wenru Wang, Yu Hao, Ying Zhu, Qingmin Kong

**Affiliations:** College of Food Engineering, Harbin University of Commerce, Harbin 150028, China; liangdong199082@163.com (D.L.); 19833708336@163.com (C.W.); 18522005754@163.com (D.L.); liuxiaofei72@163.com (X.L.); xiaoshuangcai03@163.com (S.X.); 18434260612@163.com (Y.W.); 17713345453@163.com (W.W.); haoxiaoyu1994@163.com (Y.H.)

**Keywords:** rice bran, targeted fermentation, bioactive compounds, bioavailability, functional food

## Abstract

Rice bran is a nutrient-rich agricultural by-product, and most of the bioactive compounds in it are bound and thus have poor bioavailability. Research has demonstrated that targeted microbial fermentation is a high-efficiency bioprocess for the degradation and modification of complex macromolecules to release phenolic compounds, flavonoids, dietary fibre derivatives and other new biologically active substances. Fermentation can be used to increase the antioxidant, anti-inflammatory and metabolically regulatory effects of rice bran more efficiently by changing its structure and increasing the content of active components compared with the conventional extraction method. Although some studies have investigated how to obtain suitable microbial strains and substrates, optimisation of the processing conditions for improving metabolic and functional performance has not been achieved; otherwise, other problems will still arise in the event of industrial-scale application, such as fluctuations in raw material supply, process instability, and high production costs. In the future, the integration of process analytical technology (PAT), artificial intelligence and microbial engineering will build a large-scale intelligent and controllable fermentation system. Therefore, the specific route of fermentation for valorising rice bran into high-value functional ingredients has been identified, and the scientific foundation for developing sustainable foods and nutraceuticals has been established.

## 1. Introduction

Rice bran is a primary by-product of rice milling that contains many kinds of biologically active substances, such as γ-oryzanol, phenolic acids, tocopherols, tocotrienols and dietary fibre. It quickly becomes rancid due to the activity of natural lipases and lipoxygenases on its lipids, and many phenolics are attached to cell walls or other complexes and thus poorly bioavailable. These problems have long limited the large-scale and high-value use of rice bran.

The targeted fermentation of selected microbes or simple mixed cultures can be used to address the above problems, improve the stability of the material, and achieve the controlled release and accumulation of active components by biotransformation. To ensure that the microorganisms function properly in this way, a suitable strain needs to be selected and, at the same time, process conditions and substrates should be adjusted appropriately [1]. For example, hydrolases from *Aspergillus oryzae*, such as β-glucosidase, cellulase and xylanase, can release bound phenolics in the cell wall and increase the total soluble phenolics by 289% and free ferulic acid by a factor of 52 [2].

New components are also formed by fermentation. *Aspergillus oryzae* enhances the amount of existing phenolic acids and also produces caffeic acid (probably through the hydrolysis of chlorogenic acid) and erucic acid; however, the biosynthetic pathway of erucic acid in this system is not known and may involve elongation of pre-existing fatty acids rather than de novo synthesis [2]. *Aspergillus oryzae* in solid-state fermentation can also increase the concentration of some essential amino acids, such as methionine and lysine, by hydrolysing proteins [3]. These alterations are microbe-dependent; that is to say, the enzymes and metabolic paths for different species of microbes, such as *Rhizopus oryzae*, *Monascus purpureus*, *Saccharomyces cerevisiae* and *Bacillus subtilis*, are not the same [4,5,6].

With the progress of time and other reasons, both the increase speed of microorganisms and enzyme production will change. *Aspergillus oryzae*: Phenolic release peaks at 8–10 days and then may decrease further due to additional degradation or polymerization [2]. *Saccharomyces cerevisiae*, *Bacillus subtilis* and *Lactobacillus plantarum* were solid-state co-fermented for 72 h in a 50% moisture environment with the addition of glucanase to enhance antioxidant activity [7]. Substrate pretreatment can also enhance the function of modification [3].

Targeted fermentation is used to improve the sensory qualities and boost the bioavailability of active components in rice bran for food, cosmetics and pharmaceuticals [8]. Optimised fermented defatted rice bran can be added to low-fat yoghurt to enhance the survival of probiotics, increase dietary fibre and antioxidant activity, and change texture [9]. Fermentation can be tailored for various health purposes, such as anti-obesity and anti-diabetic effects in processed black rice bran [10], or anti-diabetic products produced by fermenting rice bran–soybean mixtures with *Bacillus* species [11]. Fermented rice bran extracts regulate oxidative stress, inflammation and the immune system in other ways [12]. Polysaccharides produced by the fermentation of rice bran with *Lentinula edodes* (shiitake) can activate the activity of natural killer (NK) cells and are thus anti-cancerous [13]. Phenolic acid generated by fermentation has also shown good antioxidant, antiviral and anticancer activities, and numerous other fields have been studied continuously [14]. Metabolites of fermented rice bran in cosmetics can be used to increase the moisture content of the skin, reduce inflammation and improve the appearance of skin, slow down ageing, etc. [15]. Rice bran extract can also be used to prepare antioxidant packaging films [16].

Reviewing recent advancements in the targeted fermentation of rice bran for high-value applications, this paper will introduce relevant research by selecting microbes, modifying production processes, and liberating and modifying bioactive substances to explore whether fermentation improves the accessibility and function of rice bran components and thereby supports the development of high-value-added ingredients for food, supplements, medicine and cosmetics.

## 2. Substrate Characteristics and Fermentation Potential of Rice Bran

Rice bran is a naturally uneven material containing lipids, proteins and polysaccharides, as well as a wide range of bioactive substances such as γ-oryzanol, phenolic compounds and dietary fibres. A relatively large proportion of these bioactive molecules are not in a free and readily available state. Based on the composition analysis, most of the phenolic acids and phytosterols are covalently linked to cell wall polysaccharides or physically enclosed in macromolecular aggregates. The design of such a structure will not improve the solubility or absorption of the components by the body, nor will they be easily processed in later steps of bio-treatment. The arrangement of these bonds will determine the extent of the fermentation required.

The fermentation of rice husks is not a straightforward linear release of encapsulated metabolites from a mechanistic standpoint [17]. In fact, there is a more complex dynamic dual-layer transformation system, and degradation and biosynthesis occur together. The microbial community is degrading the fibre scaffold structure by releasing various extracellular hydrolases to break down covalently bound plant chemicals. However, this is not the end; at the same time, the microbial metabolic system also reuses the released intermediates in a specific way by means of intermediate conversion pathways and secondary metabolite assembly mechanisms. Therefore, the final products will show both a certain quantity of natural substances and qualitative changes due to depolymerization reactions, molecular rearrangements and de novo synthesis.

With the above in mind, a model of the molecule can be constructed by decomposing it; thus the reason for the mismatch between compound yield and functional activity will be clarified. For example, after fermentation, although the total quantity of phenols has doubled, so have the antioxidant and anti-inflammatory effects. Our study of *Aspergillus oryzae*-fermented wheat bran shows that breaking ester bonds and removing sugar groups often results in simpler aglycone forms that are more effective as single molecules than their original complex forms. Therefore, by modifying the structure to reduce sugar, it can more effectively perform a certain function than by adding an increased amount of sugar-soluble substances. The reason for doing this is not only for study, but also to build a scientific fermenting method free from trial and error.

The whole network of this transformation is carried out by multiple groups of microbes. Lactic acid bacteria, yeasts and filamentous fungi are all able to produce enzymes for different substrates. Fungal xylanases and cellulases begin by degrading the hemicellulose-lignin complex. Yeasts or bacteria will convert the released ferulic acid into volatile phenols or other bioactive conjugates. Metabolic labour is divided; therefore, it has been found that the final characteristics of fermented rice bran, such as enhanced immunomodulatory function and improved oxidative stability, are not the result of a single reaction. The following are the interconnected stages that result from this process: hydrolysis of structural polysaccharides, cleavage of bound phenolics, and synthesis of new secondary metabolites. A problem that needs to be solved in order to better use agricultural waste is learning how to lead the activity of this community of microorganisms.

### 2.1. Profile of Inherent Bioactive Substances in Rice Bran

Rice bran is a relatively large-scale waste product of the rice-milling industry. It has a complicated structure and many types of bioactive substances. These compounds affect both the fermentation and application of this product. The above parts are not uniformly distributed in the bran. Many of them are confined within the lignocellulosic structure or associated with cell wall polysaccharides. Therefore, the microbial enzymes are unable to access them. The amounts of the above bioactive compounds also vary among different rice varieties. Black, red and purple rice varieties generally have more and varied bioactive substances than non-pigmented ones [18,19]. The above reasons do not include how much of a particular factor is present. They also have different bran structures of and different arrangements of the components within the bran. Therefore, they will have different fermentation speeds and different functions [20].

The two kinds of bioactive components in rice bran are either fat-soluble or water-soluble. All the groups have some health advantages. The physical and chemical characteristics of these compounds affect their interaction with the bran matrix. For example, some compounds are stored in lipid bodies, some are bound to proteins, and some are linked to fibres [21]. The above attributes will affect the release, change and production of these substances during fermentation. The knowledge of the above structure can be applied to determine the food-grade characteristics and use cases of fermented rice bran. The first few groups of bioactive components and their general features are shown in Table 1.

Rice bran oil has many good compounds because of its lipophilic group. The first is γ-oryzanol, a complex of ferulic acid esters of sterols and triterpene alcohols, which has shown antioxidant and anti-inflammatory properties [22,23]. Tocopherols and tocotrienols are other types of E vitamins. Among them, the γ-forms are generally the most common and effective free-radical scavengers [22]. Recently, some studies have also revealed that pigmented rice bran contains other essential types of lipids, such as ceramide phosphate (CerP) and monogalactosyldiacylglycerol (MGDG), which are also healthy [22]. Furthermore, tocopherols and tocotrienols in the vitamin E complex are responsible for providing oxidative stability and a radical scavenging effect, and the γ-isomers in this group are the most frequent [29].

The function of a lipophilic group in fermentation depends on both the composition of the group and its position in the matrix. Many compounds are in lipid bodies or are parts of biomembrane complexes. The above forms can prevent microbial or enzymatic damage to the compound in fermentation, or cause this damage. Thus, fermentation is used to reduce lipid oxidation. Alternatively, ferulic acid esters can be used to modify the bioactivity by modifying ferulic acid esters; alternatively, other components of the matrix may be changed to alter bioactivity.

On the other hand, a wider variety of compounds are within hydrophilic groups. These are phenolic compounds, dietary fibres and proteins. The first kinds of antioxidants in rice bran are phenolic acids and flavonoids. Many of them are bound and thus cannot be used in their original form. These phenolic compounds are chemically bound to arabinoxylans in the cell walls; therefore, the end of fermentation means the breaking of these bonds [27].

The matrix of pigmented rice bran also contains a relatively large quantity of secondary metabolites, such as alkaloids and steroidal glycosides, and these brans exhibit specific properties and show a strong response to fermentation [18]. Dietary fibre also provides a physical support for bound phenolics and a fermentable substrate for microbes [27]. Proteins are also food and nutrients, and many of them are highly bioavailable bioactive peptides. Correlatedly, some have found several storage proteins and enzymes in humans that are related to antioxidant defence and other metabolic functions [18].

Rice bran is the prospect and the challenge. It has many healthy-living compounds with good prospects. However, most of the compounds are in the matrix or in forms that are difficult to release and absorb. It is not that the bioactive substances are not present; rather, they are scarce and difficult to extract in a usable form—which targeted fermentation can address. It can overcome the structural constraints of using rice bran to a certain extent. Fermentation breaks down complex polysaccharides through the disruption of molecular interactions, releasing bound or hidden compounds in an accessible and more effective form and releasing the potential energy stored in the rice bran during this process. Thus, the structure of rice bran provides the initial material for fermentation and offers an explanation as to why precision fermentation technology is now required, as shown in the following parts.

### 2.2. Fermentation-Driven Bidirectional Transformation

Rice bran fermentation is a bidirectional transformation process that degrades raw materials and produces bioactive molecules simultaneously; thus, it has been expanded beyond the limits of its original structure in recent years. Microorganisms are metabolic catalysts that degrade macromolecules and bound components into smaller, more bioavailable forms; at the same time, they synthesise new metabolites de novo; thus, the functions of rice bran can be substantially altered by this dual action and are not present in unprocessed material.

The main reason why microbial enzymes can restructure the cell wall components of rice bran and thus release bound phytochemicals is that these enzymes hydrolyse cell wall polysaccharides and other components in the structural wall structure [Table 2]. Esterases of microorganisms hydrolyse ferulate esters in the cell wall to release free phenolic acids, for example. As a result of this change, the antioxidant capacity will be significantly enhanced, as shown in the previous studies [30]. Fungal fermentation of black rice bran is known to produce free phenolic acids, but the amount of this increase varies according to the fungal strain and fermentation time, so strain-specific optimisation is required. Phytochemicals are released at the same time as the breakdown of fibre and anti-nutritional factors due to fermentation. Anaerobic fermentation of microorganisms in the rumen reduces the crude fibre content of rice bran, improves the digestibility and nutrient availability of feed and food [31]. Enzymatic hydrolysis of lignin and hemicellulose can soften the rigid structure of the bran matrix and release bound proteins and antioxidants [32]; our group has observed that the solubility of proteins in fermented samples increased by as much as 180% compared with untreated bran [32]; thus fermentation can enhance the bioavailability of nutrients for both humans and animals.

**Figure 1:**
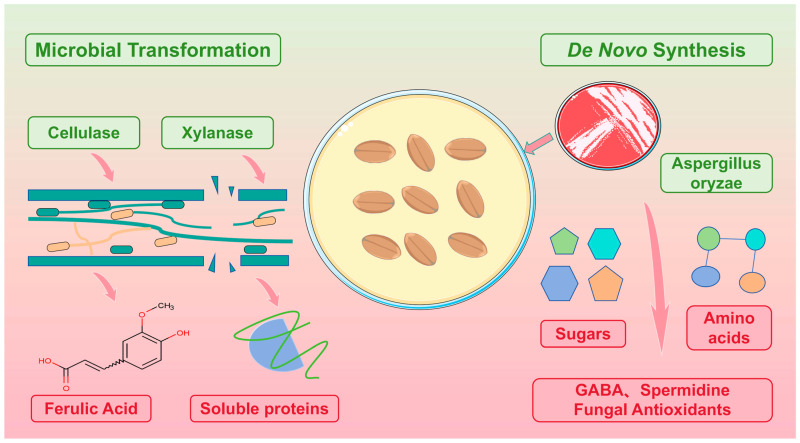
De novo synthesis by microbial transformation.

As shown in Figure 1, the two ways in which fermentation enhances the effects of rice bran work together to some extent. First, enzymatically breaking down the structure to release bound bioactive substances, such as ferulic acid; second, producing various functional metabolites through microbial de novo synthesis, including γ-aminobutyric acid (GABA), spermidine and other antioxidant compounds. How do the above processes work together to achieve a specific function? Together, they have increased the biological activities of rice bran and are likely due to a joint effect of structural breakdown and the production of metabolic substances in fermentation.

The increase in the function of fermentation may be due to an increase in compound concentration or a modification of molecular structure; both are reasonable reasons. To increase the oral absorption and efficacy of a poorly absorbed, weakly binding drug, chemical modification is performed to produce a more bioavailable and active derivative. Microorganisms produce various other substances during their metabolism, and some of these are not present in the original rice bran or are in very low amounts. Fermentation by *Aspergillus oryzae* is an example that can raise the amount of spermidine by as high as 158% and is a polyamine that promotes cell health [34]. Microbial consortia for sake production can increase GABA levels to a certain extent, and GABA is a neurotransmitter that reduces anxiety and lowers blood pressure [35]. The above cases show that molecular restructuring and new biosynthesis work together to add value, and both methods have different strengths in improving rice bran.

The biosynthetic products of these fermentation systems are not the same, and their actual effects will be influenced by both the particular strains of microorganisms and various other fermentation conditions. Solid-state fermentation with *Rhizopus oryzae*, for example, can increase the total crude protein content by more than 62% through microbial biomass accumulation [33], and edible fungus *Hypsizigus marmoreus* can produce fermentation liquids rich in new antioxidants [36] that have been applied to the development of functional food. Microorganisms can also reduce the amount of polyunsaturated fatty acids and produce free fatty acids by altering the fat content of food [Table 3]; thus, an extended shelf life of rice bran-based products can be achieved [37]. Based on the above research results, it can be determined that targeted fermentation is an excellent design-oriented bioprocess; through metabolic pathway analysis, synthetic biology and other process optimisation methods, the production of bioactive substances can be precisely controlled to enhance the functional benefits of fermented rice bran in food and animal feed.

## 3. Strategies and Mechanisms of Directed Fermentation

Fermentation can be employed to promote the production and enhance the quantity of bioactive substances in rice bran. The three reasons are: the attributes of rice bran, the power of microorganisms, and reasonable design and precise operation of the process. As shown in the previous section, fermentation involves a variety of changes that occur to the materials in rice bran due to the action of microorganisms. However, one of the problems is how to guide these changes to achieve a particular functional effect.

To overcome the inherent limitations of rice bran, directed fermentation requires a new way of thinking that deviates from improving the process by trial and error and instead designs it according to principles [39]. Fermentation can be viewed as a controllable system here. Modify one or more necessary factors in the system to alter microbial activity intentionally. Select suitable microorganisms, establish a feasible operating environment for the fermentation process (such as temperature, humidity, pH and fermentation time), and conduct pretreatment of rice bran to improve the accessibility of nutrients to microorganisms and enhance microbial activity.

The above reasons are interconnected, and together they control the release, change and formation of chemical substances in the environment. For example, the selected microorganisms are specific to certain enzymes, and these enzymes in turn determine when the microorganisms can be active [40]. At the same time, changes in the properties of rice bran (usually through pre-treatment) will alter the accessibility of the bran to enzymes and change the kind of reaction that takes place. All of the above components together form an integrated control system then, selectively reorder the naturally occurring components in rice bran and synthesise new high-value compounds with specific functions through this system.

Therefore, directed fermentation is now viewed as one of the production pathways for particular biocatalysts, and this supports [41]. Build a system for modular, programmable fermenters on this platform: in these systems, we can set the final functional results beforehand and change them by modifying the input and their connections. Finally, the above method will turn the optimisation of rice bran fermentation into an organised, function-oriented engineering process rather than a trial-and-error approach.

### 3.1. Accurate Breeding and Engineering of Microbial Strains

Select and modify suitable microbial strains for rice bran fermentation. They directly affect enzyme synthesis and thus the start of metabolic activities and the production rate of medicinal components in fermentation [42]; that is to say, the selection of a strain will not only be employed in the design of the production process but will also directly affect the actual performance of this fermentation. Strain selection also needs to be in line with process parameter optimisation, and different microbes require different levels of pH, temperature, oxygen, etc., for rice bran moisture. Based on the chosen strain, all the above factors need to be adjusted optimally.

Beneficial microorganisms are common biomatter that has been widely applied in rice bran fermentation. Therefore, they are safe, have good metabolic functions, and can improve both the nutrition and health of rice bran. Increase in the total quantity of phenolic and flavonoid compounds produced during fermentation and improve their antioxidant activity have all been found for *Lactobacillus plantarum* [43]. In the same way, certain strains of lactic acid bacteria, such as *Lactobacillus reuteri*, can alter the composition of brown rice and increase the concentration of bioactive compounds, including ferulic acid and γ-oryzanol. Therefore, the selected microbes will be in excess in the sample [44]. Strain-specific activities of yeast can grow on rice bran and, as a result, also uses it as a single source of carbon and produces different metabolites. These metabolites have increased biological activities, and ferulic acid in some rice bran varieties has been amplified [45].

Based on the above results, it can be concluded that the function of rice bran fermentation is the sum of the growth and metabolic activities of individual microbes. Various microorganisms have different catalytic functions, such as ester hydrolysis, fibre depolymerisation, protein hydrolysis, redox reactions, and the synthesis of secondary metabolites. Therefore, they will change differently in response to the same rice bran. Given the above, one can see that strain-specific directions are not generalisations of directed fermentation but targeted application of metabolic engineering for specific functions.

Therefore, the development of microbial strains for rice bran fermentation does not aim to isolate a single high-performance strain. Another issue to be solved is whether to use a single microbe with all-round catalytic abilities or a consortium of microbes that have different metabolic advantages for the desired effect [46]. The above problems are used to design microbial systems with specific functions. Different strains in the system can cooperate to degrade rice bran, convert the intermediate products, and finally produce metabolites. Therefore, the first step in building programmable fermentation systems to increase the added value of rice bran is to precisely screen and modify microbial strains.

**Figure 2:**
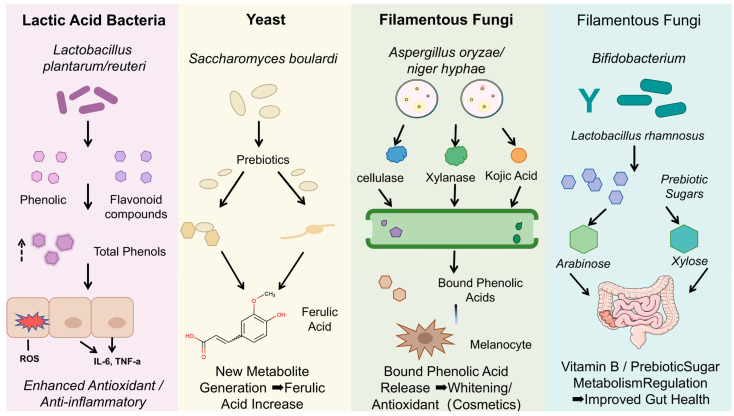
Microbial and enzymatic mechanisms for the transformation of rice bran bioactive compounds in fermentation.

Filamentous fungi, such as Aspergillus and *Mucor*, have several other strengths compared with traditional probiotics in some biocatalytic processes. They have many different kinds of enzymes. They have good adaptability to the environment of solid-state fermentation and fit well with the features of the rice bran fermentation process mentioned previously. For example, *Aspergillus niger* and Aspergillus amsterdami (both in the genus Aspergillus) can be grown on rice bran and produce cellulases and xylanases effectively. The above enzymes are responsible for breaking down the complex sugar structure in rice bran and releasing the bound phytochemicals for improved absorption [47]. Fungal fermentation can also be used for specific functions. Solid-state fermentation of rice bran and American ginseng residue with black trichoderma has increased both the content of flavonoids and ginsenoside [Figure 2]. The final product showed good tyrosinase-inhibitory activity and thus was deemed suitable for the large-scale production of a skin-whitening agent [48].

Recent developments in strain engineering have focused on designing artificial microbial communities and building function-oriented combinations of strains. The above technologies have alleviated the deficiencies of single-strain systems by using synergistic metabolic cooperation. The first type is sequential or combined cultivation of microorganisms with different functions. For example, a two-step fermentation process can be adopted: first, *Saccharomyces cerevisiae* is used, and then a series of probiotics are added to obtain different metabolites than those produced by single-step bacterial fermentation. The above experiments have shown that specific results can be achieved, such as an increase in riboflavin (vitamin B2), by design of microbial succession targeting a specific nutrient category [49]. Microbial consortia can also be designed to achieve certain functions. For example, when fermenting rice bran with a particular combination of *Lactobacillus equi*, in vitro fermentation characteristics and the microbial community structure have been regulated to improve parameters associated with gut health [50]. Similarly, through research on the mixed-culture system of *Aspergillus oryzae* and *Mucor* in rice bran fermentation, a variety of bioactive substances with stronger activities than those achieved by single-strain fermentation can be obtained [51].

Finally, according to which functions are needed and what kind of metabolism is required to realise these functions, select and modify the corresponding microorganism strains. Lactic acid bacteria and certain fungi are generally used to enhance antioxidant capacity and phenolic content, for example. Aspergillus and Penicillium species are generally selected for their protein- and fibre-degrading abilities; therefore, their industrial enzyme systems and the reduction of antinutritional factors, such as phytic acid, can be used [47]. *Rhizopus oryzae* is suitable for the production of antimicrobial substances because it can produce phenolic extracts that are highly effective against food-spoiling microorganisms and are thus suitable for applications in food preservation [52]. These strain-specific differences have also been found in metabolomics. Different microorganisms have caused various changes in amino acids, peptides, carbohydrates and vitamins through research. Fermentation increased the concentration of prebiotic sugars (arabinose and xylose) and *Lactococcus lactis* increased the amount of pyridoxine (vitamin B6), for example [49].

In short, the above results show that the selection and combination of microorganisms can alter the process to directly change the metabolic path and functional properties of rice bran fermentation. This view is consistent with the direction of subsequent process parameter control and rice bran engineering; thus, it can be used to build an all-encompassing system for the rational design of controlled fermentation processes.

The main group of lignocellulose-degrading and phenolic-releasing organisms was filamentous fungi; lactic acid bacteria were good antioxidants, anti-inflammatories, etc., that could be used for improving gut health. No single optimum strain was found, as the efficiency of strain production is significantly influenced by the fermentation medium and experimental purpose. The reproducibility of mixed-culture fermentation systems in different laboratories was relatively low, and ideal strain ratios had not been determined. A typical research deficit at the time was that no organised and standardised comparisons had been conducted systematically for single-strain and mixed-strain fermentations across multiple types of rice bran.

### 3.2. Smart Control of Fermentation Process

The second level of intelligent control for regulating the rice husk fermentation process is intelligent management of process parameters [53]. The following are general physical and chemical properties of microbes that affect their metabolic activity, nutrient utilisation rates and so forth: In terms of fermentation, conditions such as pH, temperature, water content, oxygen availability and other necessary factors do not work independently; rather, they work together to impact the rate of substrate degradation and the type and amount of bioactive compounds released. Conventional optimisation generally considers only a fixed operating condition; therefore, with alterations in the metabolic demand of fermentation over time, intelligent control can be used to adjust according to this change and address both theoretical optimisation and actual process performance.

Among the above main control parameters, moisture content is required for the start-up of all stages of microbial growth in a solid-state fermentation (SSF) system. SSF is based on a relatively stable water level to improve the accessibility of the substrate for microbes and enzymes in the lab, but this effect is very different among different strains. *Saccharomyces cerevisiae*-fermented rice bran experiments have shown that the peak of microbial activity occurs at a moisture content of 60%, and the maximum cumulative gas production is a suitable indicator of fermentation efficiency [54]. Create a relative-moisture environment to supply nutrients for decomposition and increase the porosity of the substrate; thus, complex substances are broken down: for example, at the optimal moisture content, we observed a reduction of 20.1 per cent in crude fibre and an increase of 23.1 per cent in crude protein [54]; this indicates that water can reduce the quantity of substrate for decomposition and alter the structure of decomposition by-products.

All of the following are required for enzyme activity and microbial life in a fermenter: temperature, pH and moisture content. Specifically, at the same time, a suitable temperature should be maintained in the process of lactic acid fermentation; if rice bran is used as the main nutrient source, careful control over the temperature can increase both lactic acid concentration and overall efficiency significantly [55], and such regulation is required for large-scale production. At the same time, the pH in the reaction environment is not constant and changes over the course of microbial life—gradually lower the pH due to the production of organic acid to reduce the number of pathogens and promote the growth of certain microorganisms. The first one is relatively non-sterile, so this issue is not a problem in the process [56]. How do the above parameters cooperate to keep the system stable? Research has shown that the two are required to regulate microbes more precisely and achieve the expected results of fermentation.

The factors of fermentation are not independent; they generally interact with each other and thus affect the results of hydrolysis reactions, microbial growth and metabolite transformation at the same time. The two effects work together to promote simultaneous saccharification and fermentation; at this time, enzyme-catalysed hydrolysis of cellulose and subsequent microbial acid production occur together. For example, our team has carried out research on the regulation of fed-batch culture and has shown that, by accurately controlling the supply of nitrogen and carbohydrate sources, adding rice bran can effectively alleviate substrate inhibition (a common problem in high-yield fermentation), and stable ethanol production can be achieved [57]. Thus, the control parameters need only to regulate the individual factors and coordinate metabolic pathways and optimise process timing to fully realise the potential of rice bran substrates.

Intelligent regulation can now be applied to achieve high-precision, adjustable control of the fermentation process [Table 4]. Based on the particular requirements of microorganisms and substrates, the parameters can be dynamically changed to maintain a stable and accurate state for this system. The above method is to add a real-time feedback loop in the fermentation system to increase the yield of various bio-products for people and animals, such as food and feed, as well as industrial biocatalysts [58].

**Figure 3:**
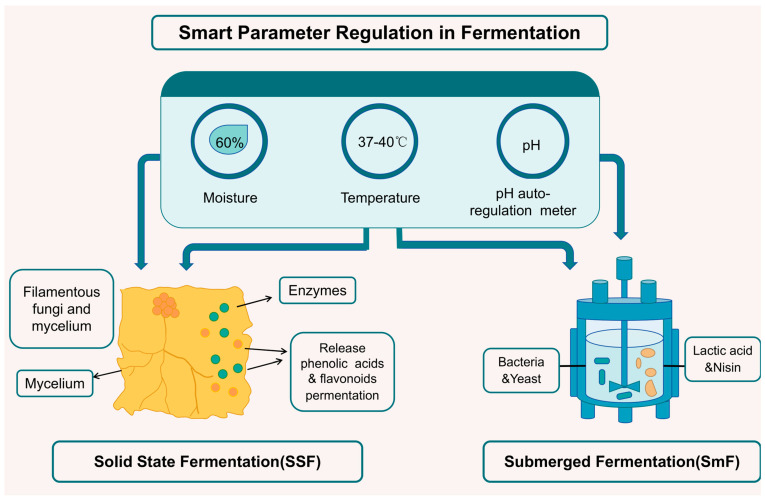
Smart parameter regulation in fermentation.

Under different environmental conditions and in different microbial communities within the fermentation system, various fermentation pathways can be selectively regulated in solid-state and submerged fermentation processes [Figure 3] such as process-level optimisation.

In the bioprocessing of rice bran, a selection needs to be made between solid-state fermentation (SSF) and submerged fermentation (SmF) to decide whether to use them; both have strengths that suit different production goals and types of microorganisms. SSF has a low water activity and is therefore suitable for the growth of filamentous fungi such as *Aspergillus oryzae* and *Neurospora sitophila* in a way that mimics natural environments. The system will be highly energy-efficient and environmentally friendly, and at the same time increase the production rate of hydrolytic enzymes and other bioactive substances. Based on our comparison experiments, SSF has increased the phenolic content of rice bran by 33.9% to 61.7%, and this particular amount is influenced by both the microbial strain and the process conditions [59]. In addition, the porous structure of the solid support can help oxygen reach the aerobic fungi and enhance their production of enzymes for the decomposition of the complex matrix in rice bran.

SmF is relatively simple, and a stable reaction system that is easy to control for pH, oxygen and temperature can be used. The above attributes are relatively suitable for SmF in bacterial fermentation and the large-scale production of microbial metabolites. Clostridium acetybutyricum can maintain a stable state and increase product recovery under SmF conditions during the continuous butanol fermentation of pretreated rice bran [62], thus reducing the difficulty of downstream processing in an industrial setting. Engineered *Lactococcus lactis* also produced streptomycin in SmF under hydrolysis conditions of rice bran and soybean meal, and its yield was relatively stable due to uniform nutrient supply and a stable process [63].

In the selection of SSF and SmF (microbial fermentation), both the efficiency of substrate utilisation and the scale-up capacity need to be considered. SSF technology has a certain advantage in the release of plant-derived bioactive compounds and is thus more suitable for the development of functional foods; on the other hand, SmF technology is more suitable for the industrial production of microbial metabolites, such as organic acids, solvents and bacteriocins. Therefore, the selected fermentation system should meet the actual needs of this research; that is, it should be suitable for laboratory-scale experiments or large-scale production, based on the specific characteristics of the microorganisms and other operational conditions of the process.

Now, the advanced control strategy will coordinate various independent variables to optimise all aspects of the fermentation process simultaneously. According to the above research, using *Rhizopus oryzae* in a rice bran fermentation system, both the size of particles and nutrient concentration (such as ammonium sulphate) influence the fermentation rate [64]; reducing the particle size increases the substrate surface for microbial consumption, and at appropriate times, high-level nutrients must also be present to sustain microbial growth. Continuously performing processing operations such as solid-state fermentation and extrusion improves both the biological activities and other functions of rice bran, and optimises the process design and control parameters to maximise the substrate value [59].

All the time during the entire process of rice bran fermentation, both the properties of the substrate (e.g., its components and structure) and the environment outside the fermenter during fermentation (e.g., temperature, pH value and oxygen content) must be taken into account. Through substrate engineering, fine-tuning of parameters, and intelligent process design, fermentation systems can unlock the hidden functional potential of rice bran and create various high-value bioactive materials for different industrial and nutritional applications from this low-grade waste product.

### 3.3. Substrate Modification and Synergistic Fermentation

Microbial selection and environmental control are the first two ways to reduce the production of undesirable substances during regulated fermentation; the structure of rice bran can be further improved in a third level to enhance the efficiency of biotransformation. Lignocellulosic materials have a lignocellulose structure, are composed of numerous macromolecules in cell walls, and all are natural impediments to microbial enzyme access and activity. In addition to addressing the structural problems above, many other factors may limit the efficiency of fermentation and biotransformation, so most generally speaking, specific pretreatment of the substrate should be included in a general fermentation plan.

Preparation of the substrate also intends to guide the metabolic path of microorganisms and thus enhance the speed and yield of fermentation [65]. Pretreatment is carried out to alter the structural and chemical features, improve the digestibility of substrates, change enzyme–substrate interactions and microbial activities, adjust metabolic pathways, etc., thereby expanding the utilisation scope of the entire potential in rice bran. Rice bran is particularly difficult to process: many of its bioactive components (such as phenolic acids, proteins and other functional molecules) are either physically encapsulated in the cell wall matrix or chemically bonded to cell components; therefore, they are not accessible to microbial enzymes and thus not available during fermentation. Targeted pretreatment of the substrate to eliminate the aforementioned barriers will liberate active components and enhance the functional performance of the final product.

To reduce the structure of rice bran and increase its specific surface area for fermentation, grinding and high-pressure water treatment (HHP) are often used as physical pre-treatment methods. The new-generation technology can now distribute nutrients and enzymes over a larger area in a more dispersed manner within a less-permeable matrix. For instance, in our first experiment, by using HHP [66] together with fermentation, both the yield and the biological activity of functional components in fermented rice bran were significantly enhanced due to the combined effect of structural disordering and enzyme activity. In short, the physical barriers have been reduced to improve the binding efficiency and accuracy of enzymes to their substrates.

On the other hand, enzyme pretreatment technology can selectively break down and precisely modify specific structural polymers in rice bran to change the fermentability of rice bran. Xylanase is a representative enzyme that hydrolyses xylan, a main component of the cell wall, into arabinose and xylose. These compounds are prebiotic and can also promote the fermentation of substrates to a certain extent [67]. Selectively degrading can enhance the supply of substrates, generate intermediate products that are then microbially degraded into metabolically advantageous prebiotics, and improve economic feasibility through increased efficiency of metabolite production. Enzyme-pretreatment technology has good precision and is therefore suitable for controlling the course of metabolism.

Exogenous pretreatment is not the only way to modify the structure; fermentation can also be employed to change the structure of the in situ substrate naturally. In fact, the special features of solid-state fermentation (SSF) are in part due to the endogenous enzymatic activities of traditional starter foods (e.g., Ropengania, Aspergillus, etc.) that produce proteases and carbotase enzymes. These enzymes gradually break down the cell wall structure and release bound nutrients [68]; this happens simultaneously with microbial growth and metabolite synthesis. In situ reactions have been observed that increase the release rate of binding molecules, boost their biological activity and function, and so on; for example, the hydrolysis of proteins into smaller, more bioavailable molecular forms [Table 5].

Substrate engineering does not have to be done in a single-substrate system; cooperative fermentation, for example, can use other supporting materials or microbial communities to create an ideal biochemical environment for rice bran and achieve novel functions. Agricultural by-products or medicinal and food crops can often be added to rice bran to enhance the nutritional value of rice bran and increase the quantity of bioactive precursors. By the synergistic enzymatic activity of *Aspergillus oryzae* and Lactobacillus helveticus, macromolecules in corn gluten meal and rice bran co-fermentation can be broken down; thus, better protein quality can be achieved and various bioactive components can be released [70]. The rational design of synthetic microbial communities (SynComs) can be used to direct metabolic flux towards desired products, such as custom-designed raw materials for aquaculture feed [71].

In addition to co-fermentation at the same time, synergy can also be achieved in a sequential or cascading manner. For example, combined co-fermentation and enzymatic hydrolysis of defatted rice bran significantly increased total phenolic and flavonoid content (by 43.59% and 55.10%, respectively), as well as in vitro and intracellular antioxidant activities, and all were better than the single treatments [69]. Co-fermentation has also been employed in the valorisation of waste within the circular bioeconomy to moderate the microbial community by co-fermenting Chinese cabbage waste with rice bran, suppress undesirable microorganisms, reduce ammonia–nitrogen production, and thus convert these wastes into livestock feed [72].

Therefore, to promote the stable establishment of a functional synergistic microbial community and support both microorganisms effectively, a suitable substrate system needs to be constructed for this co-fermentation. The internal structure of the co-substrate has an effect on the fermentation rate. For instance, different varieties of rice contain various amounts of ferulic acid (a main precursor for bio-vanillin), and therefore, choosing one of these varieties as the fermentation material may improve the yield of the following bio-conversion [73]. Mixed-substitute systems are easy-to-use nutrients for fast-growing microbes, and structurally complex carbohydrates lead to an extended release of carbon and prolonged metabolic life. Under the above conditions, cascade-upgrading technologies can be employed to produce lactic acid via fermentation of dehulled rice bran and then convert it further into biogas for high-efficiency resource utilisation [74].

In short, substrate engineering and microbial cooperation can be seen as a process optimisation problem. The substrate ratio, nitrogen source and pH of the key parameters need to be carefully regulated to guide metabolic pathways and maximise the production of target products such as bioflocculants and phenolic compounds [75,76]. The above optimisation will further enhance the function of the substrate-engineering control module and realise high-efficiency, programmable rice bran fermentation.

## 4. Fermentation-Driven Enhancement of Bioactive Substances and Functional Expansion

Based on the logical system for the design of fermentation systems, the next problem needs to be solved: how can the above ways be converted into noticeable increases in the level and function of bioactive compounds? Rice bran fermentation increases the quantity of bioactive substances in the original raw materials during this process. Modify the shape of the molecule and thus alter how easily the body can use it and what biological effects it will have. The above modifications help us observe the improved performance of fermented rice bran in a team effort. Breaking down the base material, changing metabolites, and building new substances via microorganisms are carried out in this work.

Reduce difficult-to-hydrolyse components in rice bran through molecular fermentation. The above are the cell wall components and large molecule groups. Some of the restricted plant chemicals and nutrients will be released. With a breakdown of the lignocellulose structure that contains phenolic acids, flavonoids and other active components, these compounds will be available in practical quantities. The purpose of fermentation is to release things rather than to retain them. Microbes modify the middle products in various ways to make them more effective.

Fermentation can also increase the bioavailability of the bioactive substances in the body for absorption and utilisation [77]. Reducing the size of the molecule, changing the chemical structure, and breaking the connection between the bioactive substances and the rice bran base [78] means they are more easily absorbed in the gut. The above alterations reduce the distance between having a compound lift and actually performing one. This will solve a problem of raw rice bran.

Fermentation causes changes in molecules and shapes that improve biological function at the level of function. Based on the above studies, fermented rice bran is known to have antioxidant properties and can help regulate blood sugar and fat metabolism; additionally, some other papers have demonstrated its immune-stimulatory effects. These improved functions are not always directly associated with a single compound. Therefore, shape alteration and a new mode of life are required for this activity.

We aimed aim for fermentation to be used to enhance certain functions deliberately. We select some microbes, arrange the environment for the experiment properly, and alter the contents that are added. A certain kind of control allows us to make fermented rice bran for various purposes. Functional food, health-boosting products and skincare products are typical examples. Therefore, fermentation is not solely for the purpose of increasing the value of rice bran. It is a good tool for making use-focused bio-parts.

### 4.1. Improvement of Bioaccessibility and Bioavailability

A specific method of production that can improve the absorption and utilisation of bioactive substances by the body is directed fermentation—some are also new bioactive materials; of which there is also a relatively large amount. A good application of enzymes is to break down difficult mixtures and convert some components into a state where they can be dissolved—they are the top ones. The reason is that the rigid plant cell wall and the strong binding of phenolics to fibre prevent the release of phenolics during human digestion [79].

Fermentation by microbes is also used to break down food in other ways—gut microbes that reside in the large intestine. Therefore, the large mass will be divided and the phenolic compounds that could not be extracted earlier will be released. Rice bran dietary fibre (RBDF) is a typical case; only 2.68% of the bound phenolic compounds were released during simulated gastric and intestinal digestion, but 27.57 per cent were free after colonisation fermentation [79]. This large jump shows that microbial enzyme work has helped overcome the natural barriers to obtaining plain rice bran. Table 6 shows what different bioprocessing methods do to free and use tied-up phenolic compounds (BPCs) in grain bran. List some research contents here.

Fermentation will work better if you pre-treat the base material in a smart way first. The bran frame is removed physically or enzymatically in this treatment. It facilitates the activity of microbes on the bound compounds in the next round of fermentation. Tests of highland barley bran (HBB) show the above connection. Some treatments changed how much of the tied-up phenolics were released during false digestion and fermentation [80]. Enzyme treatment of HBB freed 10.90% of the BPCs after stomach–intestinal digestion in one case. Microwave treatment showed the highest overall BPC utilisation by the body and was beneficial for gut microbes in colonisation [80]. A combined approach of pre-treatment and fermentation has reduced the amount of free bioactive compounds as much as possible.

Fermentation produces other free-floating components as well. They will also alter the chemical structure and are therefore more readily absorbed by the body and have a prolonged effect. Microbes carry out changes during fermentation to add sugar groups or break ester links. The changes will make the compounds more permeable to the cell wall. Fermented RBDF still has a good effect of inhibiting harmful free radicals. It also had the effect of blocking β-glucosidase. Therefore, it can be seen that the altered varieties have maintained or enhanced the bioactivity of fermented products [79].

Not all rises in the total phenolic content are associated with higher biological activity. The fermentation of pretreated HBB also produces a large amount of short-chain fatty acids (SCFAs), primarily butyric acid and propionic acid [80]. SCFAs are very good for gut health. They can help keep the inside of the gut in good condition. The latter will be absorbed by the colon. Thus, a good loop has been established to make the use of other food parts more efficient. Therefore, the good aspects of aimed fermentation are not limited to the liberation and transformation of bioactive substances.

### 4.2. Discovery and Identification of Novel Bioactive Substances

Fermentation of brown rice bran is also a relatively good method. It increases the amount of existing bioactive substances and produces new ones. In order to achieve the above effects, the following high-end analytical instruments will be employed. Metabolomics can identify and describe all new metabolites comprehensively. Fermentation changes the metabolic status. Microbial enzymes degrade the difficult-to-obtain components in brown rice bran to produce new functional materials.

Some research has also identified new kinds of beneficial chemicals produced by specific fermentations. *Aspergillus oryzae* fermentation of brown rice and rice bran (FBRA), for instance, has released many dipeptides. These dipeptides are not present in the unfermented material [81]. These dipeptides have biological activities and can inhibit angiotensin-converting enzyme (ACE). They are consumed more rapidly than free amino acids. Therefore, they can improve the nutritional function of fermented food [81]. The dual fermentation of rice bran by *Aspergillus oryzae* and lactic acid bacteria also yields microbial metabolites of tryptophan, such as tryptamine, at a higher concentration in the substrate. It has a strong anti-inflammatory effect on macrophages in a model. Therefore, targeted fermentation can be used to obtain metabolites with certain physiological functions [82].

The first tools for the non-target discovery of the new biological substances are high-end analytical instruments, such as liquid chromatography-mass spectrometry (LC-MS). LC-MS is used in the FBRA system to track changes in water-soluble components dynamically. It showed an increase in the free forms of vitamins B2, B6 and biotin, and also identified some new dipeptides [81]. Therefore, the problems with metabolomics are now known, and new molecules are also being discovered; nutrients previously bound to other compounds are now exposed. A large number of metabolites in the solid-state fermentation of rice bran by Aspergillus tamari were also measured. Twenty-two compounds were identified, many of which were unknown metabolites such as amines, amides, ketones, esters, sulfonic acids, hydrazides and cyclohexanes [83]. One of the compounds mentioned is 4-hydrazino-6-(4-morpholino)-N-phenyl-1,3,5-triazine-2-amine, which is a triazine derivative. Pharmacological activities of antiviral and antibacterial types were shown in this compound [83]. The type of the substrate will also affect the production of metabolites to some extent. Different metabolites are produced in different amounts by *Aspergillus tamarii* on rice bran and wheat substrates. It can be seen from [83] that contact with microorganisms and substrates is required for the release of bioactive compounds, and many studies of untargeted metabolomics lack standardised identification.

Discovery-guided isolation and purification of bioassay-active metabolites are employed to identify the source of new metabolites. For example, in a double-fermentation experiment of rice bran (FRB), it was found that the anti-inflammatory effect was stronger than that of unfermented rice bran. Therefore, we have carried out the above studies. Separation by hot water, 50% ethanol and n-hexane was carried out. Some samples were relatively less anti-inflammatory. Tryptamine was finally identified as a typical microbial metabolite [82]. In short, the combined applications of fermentation, metabolomics, bioactivity assays and compound extraction have been widely used. The aim is now to discover new functional properties of unknown peaks in chromatography. The above compounds can serve as food additives, food ingredients and pharmaceutical drugs. Table 7 shows the new bioactive substances that have been identified in fermented rice bran by targeted analysis, along with an overview of the fermentation systems, compounds, bioactivities and the supporting literature.

### 4.3. Directed Enhancement of Functionality

Other bioactive substances are produced during fermentation. To promote the individual functions of rice bran, the above way is better than adding nutrients. It can meet the various health demands of the people and produce rice bran with specific bioactivities for metabolic regulation, cardiovascular health and immune regulation.

Firstly, there is a deficiency in the antioxidant and anti-inflammatory capabilities of rice bran for the fermentation. Phenolic compounds are concentrated in the fermentation process and also change during this time. Although there are continuous reports of increased antioxidant and anti-inflammatory functions, the frequency of studies on other effects, such as antidiabetic and immunomodulatory properties, varies widely. For instance, the fermentation of Haedam and Haepum rice bran increased the total phenolic content to 156.08 mg GAE/g and enhanced DPPH radical scavenging [84]. Fermentation will also alter the kind of phenolic acid. Ferment broken rice and then bake it at 80 °C for 24 h to increase the amount of gallic acid and syringic acid. At the same time, p-catechuic acid is reduced [85]. The above are anti-inflammatory effects. Helping to prevent the disease of cancer, FBRA (*Aspergillus oryzae*-fermented brown rice and rice bran) inhibits inflammation-related cancer. It reduces colorectal cancer in ApcMin/+ mice and lung cancer in A/J mice. It can reduce the infiltration of inflammatory cells [86,87,88]. Fermentation can modify the metabolic profile of dietary fibres and bioactivities in the body related to cardiovascular and metabolic health (Table 8).

It increases the crude protein and phytochemicals of γ-aminobutyric acid (GABA) [91]. The gut microbiota degrade both soluble and insoluble fibres by fermentation. Therefore, a rise in short-chain fatty acids (such as acetic acid and butyric acid) will also be caused [94]. Fermented rice bran extract can inhibit the growth of bacteria such as *Staphylococcus aureus* and *Escherichia coli*, and is therefore a prebiotic [91]. Simultaneously prepare prebiotics and postbiotics to regulate gut microbiota and the immune system. Fermented rice bran suspensions modify the microbiota in the caecum of inflammatory bowel disease models [95].

Some of the fermented rice bran products have had an immune-modulating effect. Polysaccharides in rice bran ferments by Lentinus edodes improve the activity of natural killer cells. Fermented extracts are also effective in immune support for immunosuppressed mice [13,96]. Fermented rice bran is also for animals, enhancing immune responses in broiler chickens [97].

Many useful materials for the construction of the nervous system and cosmetics are produced by fermentation. Dihydroferulic acid (dFA) from fermented rice bran enhances the effect of ischaemia in rats [98]. The fermentation of cracked wheat decreases colour and age in cosmetics. Fermented broken rice inhibits tyrosinase and elastase, and heating increases the extent to which this inhibition occurs [85]. Fermented rice bran has inhibited the progression of cancer in the colon, oesophagus and stomach of different animal models [99,100,101].

Although there is a large amount of preclinical data on fermented rice bran, it has not yet been validated in humans [91]. Most studies to date have used in vitro and animal models; although they are informative, they cannot fully replicate the complexity of human digestion, absorption and systemic metabolism [102]. The few human-intervention trials that have studied the effects on metabolic markers, gut microbiota composition and antioxidant status have small sample sizes, heterogeneous participant demographics and inconsistent fermentation protocols, etc., which limit cross-study comparability [103]. The first clinical trials have found that the addition of fermented rice bran can help improve blood lipid levels and reduce postprandial glucose and inflammatory cytokines [91]. Based on the above research, more high-quality randomised controlled trials need to be conducted to establish the actual effectiveness and dosage–response curves of such health claims by specifying primary endpoints.

Research on all kinds of rice bran has shown that after fermentation, antioxidant and anti-inflammatory activities increase, at the same time, inhibitory factors such as phytic acid and trypsin inhibitors are reduced, and nutrient absorption and utilisation are enhanced. Some studies have reported serious hypoglycaemic effects; others have not found any, and the reasons for these differences include the degree of bran pigmentation, strain selection and fermentation time. Different strains of fungi had varying amounts of polysaccharides; therefore, an optimal fermentation condition for maximum immune-boosting effects could not be determined. The strain-dependent skin-lightening and anti-ageing effects, as well as standardised protocols for cosmetic-grade fermented bran, have not been developed. Human clinical data were scarce and inconsistent; thus, a large-scale randomised controlled trial was needed to support the health claims.

## 5. Technical Problems and Transformation Paths

Although some functions have been achieved in the laboratory by selecting fermenting rice bran, a number of technical and economic obstacles still need to be overcome before they can be implemented on a large scale in the industry. The three bottlenecks at present are related to each other: instability of the process under large-scale production, poor reproducibility among batches, and lack of standardisation for complex functional end products. Incremental optimisation through process control alone cannot solve the above problems; at this time, an organised construction of integrated and highly adaptive fermentation strategies is urgently needed that can also cope with the natural fluctuations in raw materials and fully exploit the advantages of these raw materials.

### 5.1. Key Technical Challenges in Scaling Up

Among the most difficult-to-address problems of industrialisation is the significant fluctuation in the composition of incoming raw materials. Rice bran is not homogeneous; that is to say, the concentration of active compounds, structure of fibres, and so on, are different when different varieties of rice or processing methods are used [104]. From our own work in pilot-scale solid-state fermentation, even slight differences in the lipid content or particle-size distribution of bran from different milling batches have been observed to alter the metabolic flux of microbes. Due to this variability, it is difficult to develop fermentation schemes based on fixed formulas, and the optimal parameter combination obtained for one batch of bran may not achieve the desired functional results or may be unstable when applied to another batch.

Lab-scale fermentations that use defined microbial consortia dominated by lactic acid bacteria (LAB) have been able to improve the availability of nutrients and reduce the amount of antinutritional factors such as phytic acid and crude fibre at the same time [81]. Preserving the exact metabolic results in the transition to industrial-scale vessels; however, this presents many non-linear problems. Spatial gradients of temperature, water activity, pH and dissolved oxygen will inevitably occur within large-volume solid-state beds or silage reactors; thus, heterogeneous microenvironments will be formed. In such regions, the well-organised balance of the inoculated consortium can be disturbed; as a result, niches are formed that are conducive to the growth of adventitious or spoilage-associated microorganisms, or metabolic flux is inadvertently diverted away from the desired biosynthetic pathway. Anaerobic silage fermentation is a typical case of this sensitivity: to maintain the dominance of laboratory-selected strains, a strict environmental exclusion of oxygen needs to be achieved [Table 9], but the engineering tolerances required for this uniformity increase exponentially with the expansion of the system volume [104].

Bioprocess engineering is also lacking in this study, and economically, targeted rice bran fermentation has not been promoted by policy. The development and maintenance of specific microbial strains, the application of physical or enzymatic substrate pre-treatment, energy costs for controlled fermentation, and final product downstream stabilisation are all considerable operating expenses. Therefore, the value proposition is whether the additional functional premium of the fermented ingredient can cover the accumulated costs reasonably well. For example, while inoculating specific strains of lactic acid bacteria (such as the M2 population) can significantly increase in vitro dry matter digestibility and fibre degradation indicators compared to spontaneous fermentation or untreated controls—thereby verifying process efficiency—this is done at the cost of higher operating complexity and investment [104]. At present, to achieve commercial feasibility, it is necessary to improve the economic benefits of a certain variety of wheat through production.

### 5.2. Transformation Paths for Industrialisation

Given the above problems, we need to move away from the simple, single-purpose fermentation model and build a new platform that integrates many functions. The following two synergistic strategies are expected to help bridge the gap between laboratory results and actual applications in industry.

The integration of Process Analytical Technology (PAT) can help us better observe changes in the fermentation’s internal condition and adjust production in time. A network of in-line or at-line sensors is set up to monitor key quality indicators in real time, such as pH, moisture content, exhaust gas composition and spectral fingerprints, and Process Analytical Technology (PAT) is employed to provide data support for a change from static, pre-programmed recipes [104]. Therefore, the above methods can be used to build dynamic feedback control systems that respond flexibly to changes in the substrate availability and microbial metabolic characteristics of the substrate in real time. Such adaptability is convenient and also necessary to ensure the stability of the final product in the face of the above-mentioned fluctuations in raw materials. A PAT-guided system can be employed in practice to recognise an early increase in the rate of acidification and adjust aeration or temperature automatically to return the consortium to its original metabolic state.

To expand the sense capabilities of PAT, AI and ML models are used to conduct analysis for predictive process optimisation. The above computational frameworks are good at detecting hidden patterns in high-dimensional datasets produced by multi-omics profiling (microbiome genomics, metabolomics and process variable logs), and can thus predict optimal fermentation parameters and identify early-warning signs of process deviations before product quality issues occur [104]. Besides process supervision, algorithms in artificial intelligence are now being applied to design synthetic microbial consortia more intelligently and construct strains with complementary and mutually beneficial metabolic functions for enhanced substrate conversion efficiency.

A module-based fermentation platform will be easier to extend than the design of a traditional large-scale plant. Modular architecture can be used to adjust the key elements independently, such as the composition of microorganisms, temperature fluctuations and fermentation time, and thus optimise production for a particular functional-ingredient objective. The literature shows that the above control can reduce phytic acid content in both spontaneous and laboratory-assisted fermentation; however, laboratory-assisted systems have continuously achieved additional high-value improvements in digestibility and fibre degradation that are not reliably obtained through uncontrolled natural succession [104].

By integrating the adaptability of PAT-based adaptive control, the prediction capabilities of AI, and the operational flexibility of modular systems, the industry can start to build adaptable, high-efficiency, and standardised fermentation processes that meet the high demands of commercial production and move rice bran fermentation out of the research laboratory.

Raw material fluctuations are one of the main problems for industrial production, and the addition of process analytical technology (PAT), artificial intelligence (AI) and modular fermentation should be considered for expansion. Some studies have proposed physical or enzymatic pretreatment to reduce this variation, while others have focused on the use of engineered, highly robust strains; no optimal combination has been identified. SSF had better phenolic release, but SmF offered more precise process control. Although a suitable selection could be made based on the target product and production scale, pilot-scale data that compare these industrialisation strategies under actual changes in raw materials have been lacking.

## 6. Future Prospects and Cutting-Edge Trends

Although the directional fermentation of rice husks has shown good results in the laboratory, it has not yet been implemented on an industrial scale in an economically feasible manner. Therefore, the development path of this area will no longer be one of gradual changes based on empirical data but will need to undergo a large-scale transformation into an interdisciplinary, design-led innovation system. The following subsections will present several converging trends that are redefining the scope of application for rice husk fermentation technology: the deep integration of systems biology and precision microbial engineering, the emergence of synthetic biology as a method for building custom-designed microbial chassis, and the application of these bioprocesses in a circular economy framework, all of which indicate that a new era has arrived. Now, this direction will extend the scope of research to address the industrial-scale production of high-value bio-based materials with specific functions through reasoned predictions.

### 6.1. Deep Integration of Systems Biology and Synthetic Biology

The new direction of systems biology and synthetic biology is to establish a rational, design-oriented strategy for fermentation and move away from iterative trial-and-error optimisation. Systems biology can be applied to study the metabolic interactions among microorganisms in the microbial community and how these are affected by complex rice bran materials. By combining the technologies of genomics, transcriptomics, proteomics and metabolomics, high-resolution metabolic flux maps have been constructed during fermentation to identify rate-limiting steps in the control of ferulic acid release from arabinoxylan esters or the biosynthetic pathways of γ-glutamyl derivatives and bioactive peptides [105].

The above system-level metabolic map is far from theoretical in nature; rather, it is necessary to pinpoint pathway bottlenecks and other factors that reduce the amount of product or alter molecular specificity. However, a large amount of data is still not sufficient for engineering application. At this point, synthetic biology can be used to create dynamic and implementable genetic modifications of the static metabolic map: First, build an adjustable ‘cell factory’ that can accurately replicate microbial genomes for the industrial-scale production of a specific bio-product. Accurately upregulate feruloyl esterase activity to increase the release rate of phenolic acids, inhibit competitive degradation pathways for intermediate metabolites, and even construct a novel biosynthetic pathway that is not natively present in the host strain. For example, our laboratory experiments have determined that, in comparison to wild-type strains, a moderate increase in the expression of certain carbohydrate-active enzymes in *Aspergillus oryzae* can boost the output of free hydroxycinnamic acids significantly [105].

Synthetic biology can be used to build artificial regulatory circuits to make gene switches responsive to various dynamic signals in the fermentation environment and thus improve metabolic yield in real time [106]. Furthermore, this engineering concept can also be applied to the rational design of synthetic microbial consortia for dividing metabolic tasks among various strains deliberately. A typical division of labour is to have one strain specialised in cellulose degradation to break down recalcitrant fibrous structures, releasing oligosaccharides and bound phenolics, and another engineered strain with high metabolic flux that converts these intermediates into high-value end products such as neuroactive γ-aminobutyric acid (GABA) or new phenolic conjugates [107]. Under the feedback guidance of the system at the whole-system level, such an ordered division of labour can achieve self-optimisation of the microbial community that is not possible with monoculture.

At the end of this integration is a prediction-based, model-driven fermentation design. Utilising multi-omics data as parameters, optimise through computational simulations based on genome-scale metabolic network models, and predict fermentation progress and product profiles before conducting experiments. The above computational foresight significantly reduces the research and development cycle by shifting the process development from blind screening to targeted regulation for maximising the antioxidant activity of the product or enriching specific neuroactive metabolites [105]. This integrated system is not the same as the older method of bioprocessing; a new time for precision biomanufacturing of complex rice bran systems has begun.

### 6.2. Raw Material Customization for Personalised Nutrition

The new era of personalised nutrition requires a corresponding change in raw material production methods; that is, instead of mass-produced, general-purpose supplements, functional matrices need to be developed for different physiological conditions or metabolic deficiencies. Given the above circumstances, synergistic rice bran fermentation technology has developed into a general-purpose biomanufacturing platform that can produce customised bioactive substances by adjusting microbial metabolism precisely.

Many studies have indicated that the fats in peoples’ blood are affected by fermented rice products. Research has shown that black rice bran (variety KKU ULR0381) and germinated brown rice can effectively reduce cholesterol in animal models [91]. The reasons for the above effects are consistently attributed to the increase in important phytochemicals during the fermentation process, such as γ-oryzanol, anthocyanins, γ-aminobutyric acid (GABA) and specific isomers of tocopherols [91]. The above studies have supported research on the health benefits of fermentation technology and provided practical guidance for designing particular diets for individuals with hyperlipidaemia or an elevated risk of cardiovascular disease [108]. At present, the research focus is no longer on whether fermented bran is beneficial, but rather on how to regulate the environment of fermentation to enhance a specific kind of lipid-modulating effect.

Therefore, in the face of the above situation, product modification needs to simultaneously meet the requirements for both treatment effect and safety, and bioavailability. The selected parameters are not negotiable for the design of formulations for susceptible and elderly groups. Fortunately, toxicological studies of fermented rice bran extract have shown to have a good safety record consistently: no cytotoxicity was observed in human peripheral blood mononuclear cells (PBMCs), and no adverse effects occurred in repeated-dose animal studies [91]. Therefore, the first step of safety verification must be carried out before the preparation of nutritional supplements for elderly and immunocompromised people.

The safety of fermented rice bran should first ensure that it is suitable for food, nutraceuticals and cosmetics [8]. Although there is limited toxicological data available, so far it has been clear that the extracts do not cause damage to human peripheral blood mononuclear cells (PBMCs) in vitro even at very high concentrations of the extract, far exceeding the expected amount from diet [109]. Repeated-dose oral toxicity studies in animal models have further supported this conclusion; there were no treatment-related deaths, organ lesions or haematological abnormalities, and a no-observed-adverse-effect level (NOAEL) well above the expected human intake was determined [110]. Fermentation can also be used to reduce or eliminate some components that negatively affect human health in raw rice bran, such as phytic acid, trypsin inhibitors and allergenic proteins, by combining the actions of microbial phytase and protease [111]. Therefore, the degradation has increased the bioavailability of minerals and proteins; at the same time, by eliminating potentially harmful components that could cause malnutrition or allergies in certain people, it has enhanced the product’s safety.

Fermentation is also able to break down complex macromolecules directly to provide nutrients for the older people around the world today. Based on the above results, it can be concluded that enhancing the digestibility of crude protein and selectively enriching free amino acids—such as serine, threonine and tyrosine—can promote nitrogen utilisation and stimulate muscle protein synthesis to alleviate the effects of sarcopenia [91]. It has good structural and compositional modification properties for the design of functional-ingredient carriers containing fermented rice bran.

To achieve the objective of personalisation in practice, a function-oriented design method for the fermentation process needs to be adopted. Speed up the screening of bacterial strains and set up pretreatment steps and adjusted process parameters that can satisfy the metabolic requirements for production. A typical case of this idea is the sequential fermentation strategy: first, an *Aspergillus oryzae*-type fungus is employed to enzymatically break down the substrate and release bound phenolic compounds and oligosaccharides; then, a second probiotic fermentation (such as with) is performed to convert these intermediates into all kinds of prebiotics, antimicrobials and cholesterol-reducing substances.

### 6.3. Sustainable Circular Economy Model

In the development of a particular fermentation strategy for using rice husks, we still need to aim for the goal of sustainable development and ensure that the functionalisation process meets the demands of a circular bioeconomy. The cascade resource utilisation model is a process system that can produce multiple linked value chains from a single biomass feedstock and has been selected as the main basis for this green approach.

A practical and increasingly popular implementation scheme is as follows: first, defatted rice bran is used as the substrate for solid-state or submerged fermentation to produce high-value-added functional ingredients. Extract or immobilise the target bioactive substance, then send the residual waste biomass to an anaerobic digestion facility for treatment; this material still contains a relatively high proportion of organic carbon and nutrients. The second is a treatment method that can produce biogas to supply energy for heating and electricity requirements of upstream fermentation and downstream processing. This cascade logic changes what was once considered waste into a flow of resources and fully realises the closed-loop idea of “the product of one stage is the raw material for the next stage” [74].

However, the scope of synergy is much wider than the internal optimisation of processes and includes cross-sectoral waste-resource networks. Fermented rice bran substrate is rich in carbon sources and growth factors, so it can be used as a nutrient-rich material in a complementary bioprocess. Microalgae cultivation is a typical case, and microbial bioflocculants produced by using rice bran (such as the characterised polysaccharide RBBF-C9) have shown good results in enhancing algae harvesting [75]. Such connected systems reduce the environmental impact of a single process and increase the efficiency of resource utilisation overall by using rice processing by-products as carriers for the production of other high-value biomass streams.

To build the above multi-stage integrated platforms, strict process control needs to be exercised. Small adjustments need to be made to all the parameters; that is, a higher concentration of rice bran can be used, the concentration of alkaline or acidic catalysts in the pretreatment step adjusted, and the carbon-to-nitrogen ratio modified, to increase the yield and functional activity of the intermediate product—bioflocculants [75]. In addition to optimising the above processes, an all-around techno-economic analysis (TEA) and life-cycle assessment (LCA) will be carried out; together, these two will provide robust evidence of both the economic feasibility and the considerable environmental advantages of the improved process, such as reduced greenhouse gas emissions and less wastewater pollution from nutrient recycling in the process.

Fermentation waste material can be used to produce biogas at the same time as building a self-sufficient energy source for the facility. By reducing the external energy demand of a portion of the fermentation plant, these combined facilities are less reliant on fossil fuels for power and are immune to changes in energy prices. In short, the cascade and cross-linking bioprocessing models mentioned above have extended far beyond the traditional scope of waste treatment; they are now pursuing the high goal of a zero-waste, regenerative bioeconomy and are serving as models for how targeted rice bran fermentation can foster a more sustainable and resilient food system.

### 6.4. Industrial Applications of Fermented Rice Bran in the Food, Pharmaceutical and Cosmetic Industries

Rice bran is a high-fibre, high-bioactive compound by-product of rice milling with extensive development prospects; however, due to its unpleasant taste and low digestibility before fermentation, it has not been widely applied. Microbial fermentation has altered the physicochemical properties and reduced antinutritional factors of rice bran; at the same time, it has also increased the amount of bioactive substances, thereby enhancing the application value, and is now frequently used in the food, pharmaceutical and cosmetic industries.

Fermented rice bran has been added to staple food and baked goods in the food sector to improve texture, enhance nutritional value and reduce production costs; it is used as an additive in condiments and fermented products to add flavour and shorten the fermentation period; functional ingredients for dietary fibre supplements and infant food have also been developed to meet various health demands [112]. Fermented rice bran has shown an excellent bioavailability of its active components and strong pharmacological effects in the field of pharmaceuticals; therefore, antioxidant and anti-inflammatory components have been selected as starting materials to help prevent and treat diseases caused by oxidative stress and inflammation, dietary fibres and their related factors have been employed to regulate metabolism, and auxiliary therapy for cardiovascular disease and diabetes has been provided; antitumour agents have served as additional medicinal preparations, and probiotics produced during fermentation have been used to promote the health of the intestinal tract and modulate the immune system [113]. Fermented rice bran is naturally occurring and safe and effective; thus, it has gradually been added to many cosmetics in recent years, such as skincare products, hair care items and colour cosmetics, to help moisturise the skin, fight oxidation and damage, reduce inflammation, strengthen the skin barrier, nourish hair, relieve scalp problems, and improve the appearance of makeup without causing irritation [15].

**Figure 4:**
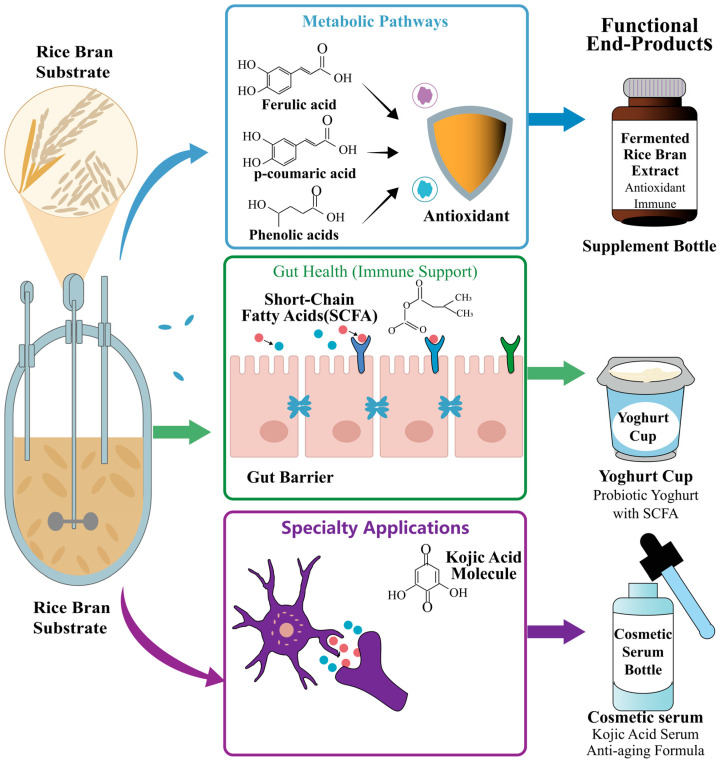
Functional transformation pathways and application-oriented outputs of targeted rice bran fermentation.

In short, fermented rice bran has promoted high-value-added use of rice-processing by-products and spurred the development of related industries. Nevertheless, uneven fermentation processes and immature large-scale production are still problems. Future work will aim to optimise the process, increase extraction efficiency, investigate the reasons in more detail, and extend the application range to make better use of resources [Figure 4].

## 7. Conclusions

This paper introduces targeted fermentation technology to upgrade rice bran into high-value bio-ingredient resources from agricultural waste. Through microbial enzyme-mediated substrate degradation and synthesis of constituents and molecules, targeted fermentation has boosted the performance of rice bran. Lactic acid bacteria, yeasts, and fungi such as Aspergillus spp. can all be used to produce various bioactive compounds under different conditions of regulation, such as pH, temperature, water and oxygen availability. Fermentation can increase or change the anti-inflammatory effect through phenolic enrichment and dietary fibre production, generating nutraceutical and cosmeceutical molecules (e.g., GABA and kojic acid). Advantages of functional ingredient design using fermentation face problems of process stability and bioactive profile uniformity. New control technology, Process Analytical Technology (PAT) and artificial intelligence are all used to solve the problem of real-time monitoring and optimisation. In the future, it is expected that systems biology, synthetic biology and data-driven modelling will be combined to create programmable microbial systems with specific bioactive characteristics. Personalised fermentation process design based on different nutritional needs and a circular economy approach. Fermented rice bran shows that compositional potential can be turned into function; thus, it is now widely used to develop next-generation bio-ingredients in a sustainable, precision-oriented bioeconomy.

Although there have been many advancements, there are still many uncertainties in the field; for example, there are many inconsistent conclusions, no unified experimental standards, and very few direct comparisons among different studies.

## Figures and Tables

**Table 1:** Main bioactive compounds in rice bran and their key features.

Compound Class	Key Representatives	Primary Sources/Notes	Content
γ-Oryzanol	Ferulic acid esters of sterols and triterpene alcohols	Major lipophilic antioxidant in rice bran oil; highest in pigmented varieties [22,23]	0.3–1.2% [24]
Vitamin E Family	Tocopherols (α, β, γ, δ), Tocotrienols (α, β, γ, δ)	Potent antioxidants; γ-tocotrienol and γ-tocopherol are often predominant [22,23]	0.05–0.25% [25]
Phenolic Compounds	Phenolic acids (e.g., ferulic, p-coumaric), Flavonoids, Anthocyanins	Wider profile in coloured rice brans; include bound forms linked to cell wall polysaccharides [18,19]	0.5–2.0% [26]
Dietary Fibre	Arabinoxylans, β-Glucan, Cellulose	Major component: insoluble bound phenolics are ester-linked to arabin oxylans [27]	The specific content varies depending on the variety and processing method [28].
Lipids & Other Bioactives	Phytosterols, Squalene, Bioactive peptides, Alkaloids, Steroidal glycosides	Lipidomic profiles are variety-specific; peptides and specific metabolites contribute to functional diversity [18,22]	15–25% [24] & 1.5–3.0% [25]

**Table 2:** Microbial transformation of rice bran components in fermentation.

Target Component	Transformation Effect	Functional Outcome	Representative Microorganisms and Literature
Cell Wall Polymers (Lignin, Hemicellulose)	Enzymatic breakdown	Liberation of bound phenolics and proteins; Reduction in crude fibre	*Aspergillus oryzae*, *Trametes versicolor* [31,32]
Phenolic Acid Esters (e.g., Ferulic Acid)	Ester hydrolysis	Increase in free phenolic acids and enhanced antioxidant activity	*Lactobacillus plantarum*, *Saccharomyces cerevisiae* [30]
Storage Proteins	Proteolytic modification	Increased protein solubility and content; Potential generation of bioactive peptides	*Bacillus subtilis*, *Aspergillus niger* [32,33]

**Table 3:** De novo synthesis of bioactive compounds in rice bran fermentation.

Bioactive Compound	Producing Microorganism	Reported Change/Outcome	Representative Literature
Spermidine	*Aspergillus oryzae*	Increased from 23.4 mg/kg to 37.0 mg/kg (content increased to 158% of original level)	[34]
γ-Aminobutyric Acid (GABA)	Fermentation consortium (for Huangjiu)	Content increased from 7.2 mg/100 g in raw rice bran to 28.7 mg/100 g after fermentation	[38]
Microbial Protein & Metabolites	*Rhizopus oryzae*	Crude protein increased from 12.3 g/100 g to 20.0 g/100 g (>62% increase); total phenolics increased from 0.8 to 2.1 mg GAE/g	[32,33]
Fungal Antioxidants	*Hypsizigus marmoreus*	Total phenolic content increased from 0.65 mg GAE/g in raw rice bran to 2.90 mg GAE/g in fermentation broth	[36]

**Table 4:** Influence of key fermentation parameters on bioactive compound profiles in rice bran.

Targeted Outcome	Key Parameter	Optimal Range/Condition	Impact on Bioactive Substances/Process	Representative Literature
Enhanced Microbial Activity & Nutrient Upgrading	Moisture Content	60% (*w*/*w*, wet basis) in solid-state fermentation of rice bran	Maximised gas production, increased crude protein (23.1%), decreased crude fibre (20.1%)	[54,59]
Phenolic & Flavonoid Enrichment	Fermentation System & Strain	Solid-state fermentation with A. oryzae or N. sitophila (30 °C, 72 h)	Increased total phenolic content (up to 61.7%) and flavonoid content (up to 53.8%)	[59]
Lactic Acid Production	Temperature Control	Optimum strain-specific (e.g., ~37–40 °C for many LAB)	Maximises lactic acid yield and volumetric productivity from rice bran hydrolysates	[55,60]
Process Stability in Non-Sterile Systems	pH	Self-induced pH drops to ~4.0–4.5 via in situ acid production during fermentation	Selectively favours lactic acid bacteria and inhibits contaminants	[57,61]

**Table 5:** Representative substrate modification strategies for rice bran fermentation.

Modification Type	Specific Method/Agent	Primary Target/Effect on Substrate	Impact on Bioactive Compound Profile	Representative Study
Physical	High Hydrostatic Pressure (HHP)	Disruption of cell wall integrity, increased porosity	Enhanced extractability and bioactivity of phenolic compounds	[66]
Enzymatic	Xylanase	Hydrolysis of hemicellulose (xylanolysis)	Release of prebiotic arabino-/xylooligosaccharides	[67]
Microbial/Enzymatic (SSF)	Traditional starters	Secretion of proteases, carbohydrases for cell wall breakdown	Increased protein extraction yield, generation of bioactive peptides	[68]
Combined Bioprocessing	Fermentation followed by enzymolysis	Sequential microbial and enzymatic degradation of complex matrix	Significant increase in total phenols, flavonoids, and antioxidant capacity	[69]

**Table 6:** Effect of various bioprocessing plans on liberation and bioavailability of bound phenolic compounds (BPCs) in cereal bran.

Substrate	Processing Strategy	Key Finding on Bioaccessibility/Bioavailability	Representative Literature
Rice Bran Dietary Fibre (RBDF)	In vitro colonic fermentation	Bound phenolic release ratio during colonic fermentation (27.57%) was an order of magnitude higher than during gastrointestinal digestion (2.68%).	[79]
Highland Barley Bran (HBB)	Microwave pretreatment followed by in vitro digestion/fermentation	Exhibited the highest BPC bioaccessibility and promoted beneficial gut microbiota (Bifidobacteriaceae, etc.) and SCFA production after 48 h fermentation.	[80]
Highland Barley Bran (HBB)	Enzyme pretreatment followed by in vitro digestion/fermentation	Liberated 10.90% of BPC after gastrointestinal digestion and 64.23% after colonic fermentation, indicating pretreatment enhances subsequent microbial liberation.	[80]

**Table 7:** Novel bioactive substances identified in fermented rice bran through targeted analytical approaches.

Fermentation System	Novel/Enriched Bioactive Substance Class	Identified Example(s)	Reported/Potential Bioactivity	Representative Literature
*Aspergillus oryzae* on brown rice & rice bran	Dipeptides	Several detected dipeptides	ACE inhibition, improved amino acid absorption	[81]
*Aspergillus kawachii* & Lactic Acid Bacteria (dual fermentation)	Tryptophan microbial metabolites	Tryptamine	Suppression of LPS-induced inflammation (Il-6)	[82]
*Aspergillus tamarii* on rice bran (solid-state)	Diverse novel metabolites	Amines, amides, sulfonic acids, hydrazides, triazole derivatives (e.g., 4-hydrazino-6-(4-morpholinyl)-N-phenyl-1,3,5-triazin-2-amine)	Potential antiviral, antimicrobial, and other pharmacological activities	[83]

**Table 8:** Directed enhancement of metabolic and cardiovascular health functions by rice bran fermentation.

Targeted Function	Key Bioactive Components/Effects	Evidence from Fermented Rice Bran Studies
Hypoglycaemic/Anti-diabetic	α-Amylase & α-glucosidase inhibition; Improved glucose response	Bioactive flavonoids from black rice bran computationally validated as potent enzyme inhibitors [89]; Cyanidin-3-glucoside and 6′-O-feruloylsucrose identified as novel α-glucosidase inhibitors from black rice bran [90]; Anti-diabetic effect observed for material fermented from rice bran and soybean [11]
Hypolipidaemic/Anti-obesity	Reduced weight gain, serum cholesterol, triglycerides; Modulation of lipoproteins	Bioprocessed black rice bran combined with green tea extract reduced weight gain by 67% in high-fat-diet mice and showed positive trends in serum glucose, insulin, and lipoprotein levels [10]; Fermented rice bran extracts demonstrated hypocholesterolemia effects in vivo [91]
Cardiovascular Health	Blood pressure-lowering activity; Improved oxidative stress and mitochondrial function	Blood pressure-lowering activity reported for fermented rice bran [92]; Ferulic acid from rice bran improved oxidative stress and mitochondrial biogenesis/dynamics [93]

**Table 9:** Key technical challenges in scaling up targeted rice bran fermentation.

Challenge Category	Specific Issue	Impact on Targeted Fermentation	Supporting Evidence from Literature
Raw Material Inconsistency	Variable composition of bioactive precursors (e.g., phenolics, fibres) and anti-nutrients (e.g., phytic acid)	Leads to batch-to-batch variation in fermentation kinetics and final bioactive compound profile, hindering standardisation.	Study uses standardised rice bran batches; real-world application faces inherent variability [104]
Process Control & Scalability	Maintaining uniform environmental conditions (moisture, pH, anaerobiosis) in large-scale solid-state or silage systems	Risk of spoilage, inconsistent microbial activity, and failure to replicate lab-scale bioactive enhancement efficacy.	Process requires precise anaerobic conditions for silage fermentation to succeed [104]
Microbial Consortium Stability	Ensuring dominance and metabolic consistency of starter cultures (e.g., LAB) against indigenous microbiota over extended periods	Uncontrolled microbial succession can degrade target bioactives or produce off-flavours/toxins, compromising product safety and functionality.	LAB addition (M2) showed superior and consistent results in improving IVDMD and reducing CF compared to spontaneous fermentation (M1) [104]
Product Standardisation & Characterisation	Defining and quantifying the complex mixture of activated/novel bioactives (phenolics, peptides, etc.) for consistent health claims	Lack of standardised analytical protocols and biomarkers for “activated” states makes quality control and regulatory approval difficult.	Study measures gross parameters (IVDMD, CF, PA); targeted fermentation for specific molecules requires more sophisticated, real-time monitoring.

## Data Availability

No new data or processed data were created in this paper. Data sharing is not available for this paper.
